# Clinical and Histopathological Features of Renal Maldevelopment in Boxer Dogs: A Retrospective Case Series (1999–2018) †

**DOI:** 10.3390/ani11030810

**Published:** 2021-03-13

**Authors:** Maria Alfonsa Cavalera, Floriana Gernone, Annamaria Uva, Paola D’Ippolito, Xavier Roura, Andrea Zatelli

**Affiliations:** 1 Department of Veterinary Medicine, University of Bari, 70010 Valenzano, Italy; mariaalfonsa.cavalera@uniba.it (M.A.C.); floriana.gernone@uniba.it (F.G.); annamaria.uva@uniba.it (A.U.); 2 Veterinary diagnostic Lab ACV Triggiano, 70019 Triggiano, Italy; pdi.cvp@gmail.com; 3 Hospital Clínic Veterinari, Universitat Autònoma de Barcelona, 08193 Bellaterra, Spain; Xavier.Roura@uab.cat

**Keywords:** kidney, renal maldevelopment, immature glomeruli, proteinuria, inheritance, canine, Boxer

## Abstract

**Simple Summary:**

This study describes clinical findings in Boxer dogs with renal maldevelopment and proposes a possible mode of inheritance. Medical records of 9 female Boxer dogs, older than 5 months and with a clinical diagnosis of proteinuric chronic kidney disease prior to one year of age, showed the presence of polyuria and polydipsia, decreased appetite, weight loss, lethargy and weakness in all affected dogs. Common laboratory findings were proteinuria and diluted urine, non-regenerative anemia, azotemia, hyperphosphatemia, hypoalbuminemia and hypercholesterolemia. Histopathology of the kidneys identified the presence of immature glomeruli in all dogs. In 7 out of 9 related dogs, the pedigree analysis showed that a simple autosomal recessive trait may be a possible mode of inheritance. Renal glomerular immaturity should be suspected in Boxer dogs with a history of polyuria, polydipsia, decreased appetite, weight loss, lethargy, weakness and proteinuria. A prompt diagnosis of renal maldevelopment, potentially hereditary, may help to evaluate if relatives of the affected dogs might be at risk, thus assisting clinicians in reaching an early diagnosis. A routine clinical renal screening evaluation in this breed, especially when this disease is suspected, should be strongly recommended.

**Abstract:**

Renal maldevelopment (RM) has been proposed to replace the old and sometimes misused term “renal dysplasia” in dogs. Although renal dysplasia has been described in Boxers, hereditary transmission has only been hypothesized. This study reports clinical and renal histological findings in Boxer dogs with RM, proposing a possible mode of inheritance. Medical records of 9 female Boxer dogs, older than 5 months and with a clinical diagnosis of chronic kidney disease prior to one year of age, were retrospectively reviewed. Polyuria and polydipsia (PU/PD), decreased appetite, weight loss, lethargy and weakness were described in all affected dogs. Common laboratory findings were proteinuria, diluted urine, non-regenerative anemia, azotemia, hyperphosphatemia, hypoalbuminemia and hypercholesterolemia. Histopathology of the kidneys revealed the presence of immature glomeruli in all dogs, which is consistent with RM. In 7 related dogs, the pedigree analysis showed that a simple autosomal recessive trait may be a possible mode of inheritance. Renal maldevelopment should be suspected in young Boxer dogs with a history of PU/PD, decreased appetite, weight loss, lethargy, weakness and proteinuria. Due to its possible inheritance, an early diagnosis of RM may allow clinicians to promptly identify other potentially affected dogs among the relatives of the diagnosed case.

## 1. Introduction

Renal dysplasia is a frequently described congenital disease in veterinary medicine [1], defined as a disorganized development of the renal parenchyma due to an abnormal differentiation [2,3,4]. Distinct histopathological features characterize renal dysplasia, such as the presence of metaplastic cartilages, primitive ducts and lobar disorganization without inflammation [2,3,4]. Other congenital disorders described in the veterinary literature are juvenile or familial renal disease and hereditary nephropathy [4,5].

Recently, the term “renal maldevelopment” (RM) has been proposed to replace the old and sometimes misused term “renal dysplasia” in order to describe a type of juvenile-onset chronic kidney disease (JOCKD) [5]. The dominant histological feature of RM is the presence and persistence of fetal/immature glomeruli [5].

In dogs affected by familial glomerulopathies, clinical signs can be observed at different ages ranging from a few weeks to several years, the most common being lethargy, anorexia, vomiting, weight loss, and polyuria and polydipsia (PU/PD) [5]. Depending on the percentage of either normal or abnormal nephrons, the disease may be staged as severe, moderate or mild [3]. In severe forms (i.e., more than 25% of glomeruli affected in total), most of the affected dogs die of renal insufficiency at between 3 and 6 months of age; alongside anemia, azotemia and PU/PD, failure to grow is a prominent feature. Dogs affected by moderate forms (i.e., 10–25% of glomeruli affected in total) usually live for 1–3 years, during which time they develop progressive chronic kidney disease (CKD); excessive water intake, azotemia and stunted growth are frequent findings. In the presence of mild forms (i.e., less than 10% of glomeruli affected in total), dogs may develop renal failure in adulthood or else remain clinically normal; excessive water intake represents the hallmark of the clinical history [3]. Proteinuria of different degrees is identified regardless of the form in the majority of cases, whereas laboratory findings such as non-regenerative anemia, azotemia, hyperphosphatemia, hypoalbuminemia, hypercholesterolemia and diluted urine are less frequent [4,6].

Different types of canine inherited renal diseases have been described in the Bull Terrier [7], Cocker Spaniel [8,9,10,11,12], Samoyed [13,14], Shih Tzu [15], Soft Coated Wheaten Terrier [16,17], Navasota [18], English Springer Spaniel [19] and Bernese Mountain Dog [20,21]. Canine inherited renal diseases mostly affecting the glomeruli have been reported in the Doberman Pinscher [22,23], Newfoundland [24] and Bullmastiff [25]. Although renal dysplasia has been described in Boxers, scant data on RM are available and hereditary transmission has only been hypothesized [26,27,28,29]. Therefore, this study aims to describe clinical aspects of a case series of 9 female Boxer dogs with RM, and to propose a potential mode of inheritance in this breed.

## 2. Materials and Methods

Medical records from Boxer dogs, of any sex, which were clinically evaluated between January 1999 and December 2018, were retrospectively collected. Dogs were considered eligible for this study if older than 5 months and with a diagnosis of CKD prior to one year of age. For each dog enrolled, clinical examination, complete blood count (CBC), coagulation profile (including activated partial thromboplastin time, prothrombin time and fibrinogen concentrations), serum biochemical profile, complete urinalysis (dipstick, urine specific gravity, urine sediment and urine protein-to-creatinine ratio (UPC)) from urine collected by cystocentesis, abdominal ultrasound examination and histological examination of kidney biopsy collected under ultrasound guidance were available. Renal percutaneous echo-assisted biopsies (Tru-Cut biopsy, 18 Gauge) were carried out under general anesthesia from the cranial pole of the left kidney, and tissue samples were processed, as previously described [30]. Sections of each renal biopsy specimen were stained with hematoxylin and eosin (H&E), periodic acid–Schiff (PAS), Goldner’s trichrome, methenamine silver and Congo Red stains. The PAS stain was used to best identify the integrity of the basement membrane, detect locations of deposits, and delineate crescents from the underlying compressed glomerular tuft. The trichrome stain was used to highlight areas of fibrosis or sclerosis, fuchsinophilic deposits, and duplication or thickening of the basement membrane. The methenamine silver stain was used to outline basement membranes of the tubules and glomeruli and areas of sclerosis, enable the best assessment of severity of overall renal damage, and highlight abnormalities in glomerular basement membranes (GBMs) such as spikes and duplication. The Congo Red stain was used to detect deposits of amyloid. Sections of biopsy specimens were considered adequate for histological examination when they contained at least 5 glomeruli/section.

When available, pedigree information was used to identify a potential inherence relationship between the dogs included in the study.

## 3. Results

Records of 67 Boxer dogs affected by CKD and older than five months were retrospectively reviewed. Nine female dogs (7 intact and 2 spayed) aged from 6 up to 79 months at the time of admission accomplished all the criteria and were enrolled in this study. Fifty-eight dogs were excluded because of a lack of histological examination and/or incomplete laboratory exams (*n* = 45) or final diagnosis of CKD after 12 months of age (*n* = 13).

### 3.1. Anamnesis and Clinical Findings

All dogs included had PU/PD, decreased appetite, weight loss, lethargy and weakness. Occasional episodes of vomiting were noted in 6 dogs, and stunted growth was evident in 3 dogs (#1, #3 and #4) (Table 1). Systolic blood pressure (BP) was available in 5 of 9 dogs and, according to the International Renal Interest Society (IRIS) staging system [31], all of them had severe hypertension (Table 1). Clinical history allowed us to exclude conditions such as urinary tract infections or toxin exposure in the first five months of life.

### 3.2. Laboratory Analyses

Based on CBC, all dogs showed non-regenerative anemia (hematocrit 20–29%, hemoglobin 8.3–10.2 g/dL), azotemia, hyperphosphatemia and hypercholesterolemia (Table 1). Severe hypoalbuminemia, with albumin levels <2.0 g/dL, was noted in 7 dogs. One dog (dog #3) developed hypoalbuminemia 6 months later, at the time of euthanasia. All dogs had diluted urine and proteinuria, according to the IRIS staging system [31] (Table 1). Considering that 4 dogs lived in an endemic area for leishmaniosis, an immunofluorescence antibody test for detection of anti-*Leishmania* antibodies was performed, and all these dogs tested negative.

### 3.3. Ultrasonographic Findings and Biopsy

Based on ultrasonography, the kidneys had normal size and shape (*n* = 7) or were considered slightly smaller than normal (*n* = 2). In all dogs, echogenicity of the cortex and medulla was increased to various degrees, cortico-medullary definition was decreased, and focal lesions of the renal parenchyma were not identified. Mild ectasia of the renal pelvis was present in all dogs but in the absence of urolithiasis. Pulsed-wave Doppler analysis of renal arcuate arteries, performed in non-sedated dogs, showed normal resistivity index (IR < 0.72) in all of them.

### 3.4. Histopathology

**Figure 1:**
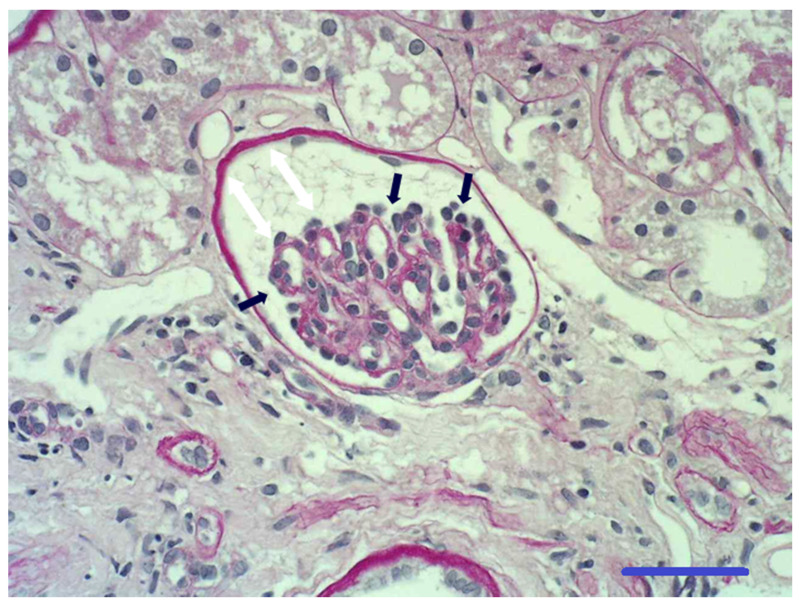
Photomicrograph of glomerulus from a section of a renal biopsy specimen. The glomerular capillary tuft is small and incompletely developed (it contains only a few small capillaries with open lumens). There is prominence of the visceral epithelial cells located on the outer contours of the glomerular capillaries (black arrows). The glomerular capillary tuft does not fill its Bowman’s capsule (white arrows), which is small without being enlarged. The Bowman’s capsule is of normal thickness and has a few parietal epithelial cells lining its inner surface. Periglomerular fibrosis is absent. Periodic acid–Schiff stain 400×: bar 50 µm.

Histopathological examination of the renal biopsy specimens identified asynchronous differentiation of nephrons with the presence of immature glomeruli (not less than 20%), which were small in size and poorly capillarized, with increased lobulation, and without cystic dilation of the Bowman’s capsule in all dogs. The visceral epithelium lining the capillary of the glomerular tuft also had an increased thickness (Figure 1). Congo Red was negative for amyloid deposits. In some areas, the interstitium was expanded by collagenous matrix. Interstitial inflammatory reaction, which is a common feature of RM, with lymphoplasmacytic infiltration, was seen in all dogs. The tubular basement membranes were of normal thickness, and some tubules surrounded by collagenous matrix were degenerate and atrophic. No other histopathological lesions were identified under light microscopy.

### 3.5. Therapy and Survival Time

Out of 9 dogs enrolled, 7 were euthanized due to the presence of severe clinical signs secondary to uremia and a fatal prognosis of the disease. Two out of 9 dogs (#3 and #6), which had proteinuria, hyperphosphatemia and azotemia, received a renal commercial prescription diet (Hill’s Prescription Diet Canine k/d^®^, Hill’s Pet Nutrition Inc, Topeka, Kan) and enalapril (Enacard^®^, Merial Italia SpA, Milano, Italy) (0.5 mg/kg *q* 12 h PO). Dog #6 was severely hypertensive and also received amlodipin (Norvasc^®^, Pfizer Manufacturing Deutschland GmbH, Illertissen, Germany) (0.15 mg/kg *q* 24 h PO). These two dogs (#3 and #6) were euthanized 4 and 6 months after the final diagnosis, respectively (Table 1). Finally, the median age at time of euthanasia was 16 months (from 6 to 79 months).

### 3.6. Pedigree Analysis

**Figure 2:**
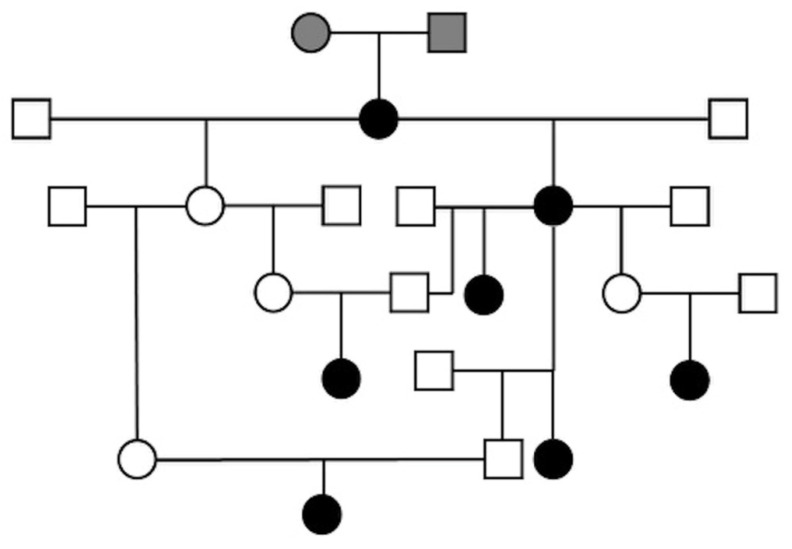
Composite pedigree of 7 female Boxer dogs affected by glomerular immaturity. The affected dogs had a common female and male mating (gray filled-in circle and square, respectively), shown at the top of the pedigree. Black circle—affected female; open square—untested male; open circle—untested female.

Pedigree information was available for 7 out of 9 dogs registered in the Italian Kennel Club, and a composite pedigree was created showing familial relationships (Figure 2). Unfortunately, pedigrees were not available for the remaining two dogs. All related dogs had a common female and male mating (gray filled-in circle and square, respectively), shown at the top of the composite pedigree (Figure 2). All affected dogs in our series were females and, based on the anamnesis, five of them were born from clinically healthy parents. This result could be consistent with a potential simple autosomal recessive mode of inheritance of RM in Boxer dogs.

## 4. Discussion

This study describes a case series of 9 female Boxer dogs affected by RM and proposes, for the first time, a potential mode of inheritance of glomerulopathy in this breed.

A case series of Boxer dogs affected by renal dysplasia was previously reported by Chandler et al. [28]. The main differences between the previous report and our study are based on sexual representation, presence of urinary incontinence and prevalence of proteinuria. Both sexes were represented in the previously published report, but with a higher prevalence of renal dysplasia in females; on the contrary, we identified only female dogs affected, although this occurrence could be incidental. Urinary incontinence, not described in the present study, was reported in 36 out of 37 dogs in [28]. Furthermore, all dogs in the present study had proteinuria, compared with 25 out of 37 in the previous paper [28]. Moreover, the authors of [28] performed kidney biopsies only in 9 of 37 dogs, and the predominant histopathological findings included pericapsular and interstitial fibrosis, dilated tubules, sclerotic glomeruli and dystrophic calcification. In the present study, all dogs underwent kidney biopsy, and the main histopathological finding was consistent with RM, being glomeruli with an immature aspect [5].

Glomerulocystic atrophy, mainly characterized by the presence of small and compressed glomerular tufts and dilation of the Bowman’s capsules, has been previously observed in Boxer dogs [5]. In our population, there was no evidence of cystic dilation of the Bowman’s capsule.

Another paper reported a case series of 7 young Boxer dogs, affected by kidney disease and with histological kidney lesions compatible with chronic pyelonephritis, associated with chronic tubulointerstitial inflammation of various degrees [29]. Immature glomeruli were found in 6 out of 7 patients [29]. Although the authors hypothesized a possible inherited origin for renal lesions in 6 of the enrolled dogs, since the pedigrees confirmed genetic relationships, they finally proposed that the end-stage kidney disease in this young Boxer dog series was most probably the result of chronic pyelonephritis, caused by vesico-ureteral reflux, predisposing to ascending infection [29]. In contrast, in the present study, glomerular immaturity was diagnosed in all dogs and neither vesico-ureteral reflux nor pyelonephritis was identified or suspected, based on anamnesis, clinical history, physical examination, laboratory and ultrasonography results, and histological evaluation of the kidney.

The familial relationship among 7 out of 9 enrolled dogs, which shared a common female and male ancestor, suggests a simple autosomal recessive mode of inheritance of RM in Boxer dogs, as previously hypothesized [29]. Similarly, the examination of the pedigrees of 11 related Bullmastiff dogs affected by glomerular disease yielded evidence supporting an autosomal recessive mode of inheritance of glomerulonephropathy in this breed [25].

The main limitations of the present study consisted in the small sample size and in the lack of use of electron microscopy to detect renal lesions clinically characterized by proteinuria, such as minimal change disease. However, conventional light microscopy is considered to be a well-proven method to detect immature glomeruli [5]. As stated by Cianciolo et al. [5], diagnoses of RM can be made based on histological evaluation together with the clinical history, and other renal diseases in this group of Boxer dogs were considered unlikely. Moreover, renal organogenesis is a complex process, and the developing kidneys (which continue to develop during the first 3 months of life) might be harmed, and their further development disrupted, by non-genetic diseases (e.g., urine reflux, infections, toxins) both in utero and in the neonatal period. Therefore, a further limitation of the study may be the lack of information related to pregnancy. However, conditions such as infections and exposure to toxins able to alter the development of glomeruli in the post-natal period were excluded through the medical history evaluation.

Furthermore, another limitation of this study was the lack of information on all littermates of affected dogs (*n* = 2) to allow an accurate estimation of segregation ratios. Therefore, an autosomal recessive mode of inheritance of RM in our breeding line of Boxer dogs could be considered a working hypothesis, although a more complex mode of inheritance could also be possible. Future studies will be needed to detect the underlying molecular abnormality responsible for the development of the inherited glomerulopathy in Boxer dogs herein described.

## 5. Conclusions

An early diagnosis of potentially hereditary RM may help to evaluate if relatives of the affected dogs might be at risk, thus assisting clinicians in reaching an early diagnosis of renal disease. Screening tests of a population of Boxer dogs considered at risk because of a familial history of renal proteinuria and the onset of CKD prior to one year of age should be highly recommended. Based on the present case series, an initial diagnostic approach based on a screening test using a urine dipstick could be suggested, as in the case of a negative result, the presence of proteinuria would be excluded, regardless of the urinary gravity [32,33].

## Figures and Tables

**Table 1:** Clinical and laboratory findings of the 9 female Boxer dogs affected by glomerular immaturity.

Dog #	Age	BUN mg/dL (RR: 8.0–20)	Creatinine mg/dL (RR: 0.5–1.4)	IRIS Stage	Albumine mg/dL (RR: 2.7–3.8)	Phosphorus mg/dL (RR: 2.5–6.0)	UPC	SAP mmHg
1	06	68	5.6	4	1.6	8.9	5.6	185
2	09	56	4.1	3	1.7	9.4	2.8	ND
3	11	46	3.4	3	2.8	7.2	2.9	ND
4	13	89	4.8	3	1.8	9.3	3.7	ND
5	16	138	6.9	4	1.9	8.4	4.8	190
6	21	56	4.1	3	3.0	6.8	1.9	190
7	29	156	6.9	4	1.4	12.3	3.4	ND
8	71	120	6.3	4	1.6	6.7	2.5	205
9	79	78	4.2	3	1.8	6.5	2.4	200

Age—age at the time of admission expressed in months; RR—reference range; BUN—blood urea nitrogen; IRIS—International Renal Interest Society; UPC—urine protein-to-creatinine ratio; SAP—systolic arterial pressure; ND—not determined.

## Data Availability

The data presented in this study are available on request from the corresponding author.
